# Hydroxyapatite Nanoparticle Modification of 3D-Printed Crown Resin: Effects of Concentration on Surface Roughness and Vickers Hardness After Thermocycling

**DOI:** 10.3390/jfb17050223

**Published:** 2026-05-02

**Authors:** Elif Koç, Dalndushe Abdulai, Oyun-Erdene Batgerel, Oktay Yazıcıoğlu, Raghib Suradi, Mehran Moghbel

**Affiliations:** 1 Department of Prosthodontics, Faculty of Dentistry, Biruni University, Merkezefendi Mahallesi, G/75 Sokak, 1-13 (M.G.), Zeytinburnu, Istanbul 34015, Turkey; 2 Department of Restorative Dentistry, Faculty of Dentistry, Biruni University, Merkezefendi Mahallesi, G/75 Sokak, 1-13 (M.G.), Zeytinburnu, Istanbul 34015, Turkey; 3 Department of Restorative Dentistry, Faculty of Dentistry, Istanbul University, Beyazıt Square (Beyazıt Meydanı) Fatih, Istanbul 34126, Turkey; 4 Department of Pediatric Dentistry, Faculty of Dentistry, Biruni University, Merkezefendi Mahallesi, G/75 Sokak, 1-13 (M.G.), Zeytinburnu, Istanbul 34015, Turkey

**Keywords:** dental materials, additive manufacturing, hydroxyapatite nanoparticle, surface roughness, Vickers hardness, thermocycling

## Abstract

Background: This in vitro study evaluated the effect of hydroxyapatite nanoparticle (nano-HAp) incorporation on surface roughness and Vickers hardness of a 3D-printed crown resin after thermocycling. Methods: Disk-shaped specimens (*N* = 84) were modified and fabricated with 0%, 1%, 2%, and 3% nano-HAp. Surface roughness (Ra) and Vickers hardness (VHN) were measured before and after thermocycling (5000 cycles). Surface morphology was qualitatively assessed using FE-SEM. Data were analyzed using two-way mixed-design ANOVA (α = 0.05). Results: Thermocycling increased surface roughness and reduced hardness in all groups. Ra values were highest in the 3% nano-HAp group after thermocycling (1.16 ± 0.47 µm). Baseline Vickers hardness differed significantly among nano-HAp concentrations, and hardness decreased after thermocycling in all groups; however, the 3% nano-HAp group exhibited the highest post-thermocycling hardness values (24.66 ± 1.51 VHN), which should be interpreted in the context of its higher baseline hardness. FE-SEM observations suggested increased surface irregularities with higher nano-HAp concentrations after thermocycling. Conclusions: Nano-HAp incorporation influenced both surface and mechanical properties, with 3% concentration showing higher hardness after aging but increased roughness.

## 1. Introduction

The fabrication of dental restorations using computer-aided design and computer-aided manufacturing (CAD/CAM) technologies has become an integral part of modern dentistry, with additive manufacturing (three-dimensional [3D] printing) increasingly adopted for the production of definitive restorations, particularly permanent crowns [1,2]. Compared with conventional subtractive techniques, additive manufacturing offers advantages such as reduced material waste, improved cost efficiency, and the ability to fabricate complex geometries with high precision [2,3]. Despite these benefits, concerns remain regarding the mechanical performance and long-term stability of additively manufactured permanent crown resins.

For clinical success, additively manufactured permanent crown resins must exhibit adequate surface and mechanical properties, particularly surface roughness (Ra) and hardness, which directly influence wear resistance, plaque accumulation, and overall material longevity [4,5]. Ra values exceeding approximately 0.2 µm have historically been associated with increased bacterial adhesion and plaque retention [6,7]. However, this threshold is primarily derived from conventional materials, and its applicability to additively manufactured resin surfaces, which may exhibit distinct topographical characteristics due to layer-by-layer fabrication, remains to be fully clarified. Concurrently, Vickers hardness (VHN) is a critical indicator of resistance to surface deformation and wear, and is strongly influenced by filler characteristics, filler–matrix interactions, and the integrity of the polymer network [4,5]. Previous studies have reported that additively manufactured resins often exhibit lower hardness compared with CAD/CAM milled materials, highlighting the need for targeted material optimization strategies for definitive crown applications [4,5].

Artificial aging methods, such as thermocycling, are widely used to simulate intraoral conditions by exposing materials to repeated thermal fluctuations and hydrolytic stress [3,4]. Thermocycling protocols involving 5000 cycles are commonly considered to approximate several months of clinical service, making them a clinically relevant approach for evaluating changes in surface roughness and hardness over time. However, the response of additively manufactured permanent crown resins to such aging conditions remains inconsistent in the literature [8,9,10].

In an effort to improve the performance of additively manufactured permanent crown resins, nanoparticle incorporation has emerged as a promising material modification strategy. Nanoparticles possess high surface area and unique physicochemical properties that may enhance mechanical behavior and interfacial bonding within resin matrices [11,12,13,14,15]. Among these, nano-hydroxyapatite (nano-HAp) has attracted attention due to its nanoscale particle size and potential to influence filler–matrix interactions and stress distribution within polymer networks [16,17,18,19,20]. These characteristics suggest that nano-HAp incorporation may affect both surface roughness and hardness by modifying particle dispersion and interfacial integrity within additively manufactured resins. However, most existing studies have focused on conventionally processed materials, and their findings cannot be directly extrapolated to additively manufactured permanent crown resins due to differences in fabrication methods and polymerization mechanisms [11,12,13,14,15,21,22,23,24].

Despite these potential advantages, nanoparticle incorporation may also present challenges. Inadequate dispersion, particle agglomeration, and weak filler–matrix bonding can adversely affect mechanical properties and may even increase surface roughness. These limitations are particularly relevant for additively manufactured resins, where layer-by-layer fabrication and polymerization dynamics may further influence nanoparticle distribution and interfacial integrity.

Although nano-HAp has been widely investigated in conventional dental materials and broader biomedical applications [21,22,23,24], its incorporation into additively manufactured permanent crown resins remains insufficiently explored, especially in relation to surface roughness and hardness after artificial aging. To the authors’ knowledge, studies addressing the combined effects of nano-HAp incorporation on both surface roughness and Vickers hardness of 3D-printed permanent crown resins following thermocycling are limited. Therefore, the aim of this in vitro study was to evaluate the effects of nano-HAp incorporation on the surface roughness (Ra) and Vickers hardness (VHN) of an additively manufactured permanent crown resin after thermocycling. The first null hypothesis was that nano-HAp incorporation would not significantly affect surface roughness (Ra) or Vickers hardness (VHN). The second null hypothesis was that thermocycling would not significantly influence Ra or VHN. The third null hypothesis was that there would be no interaction between nano-HAp incorporation and thermocycling on Ra and VHN.

## 2. Materials and Methods

### 2.1. Study Design and Sample Preparation

**Figure 1:**
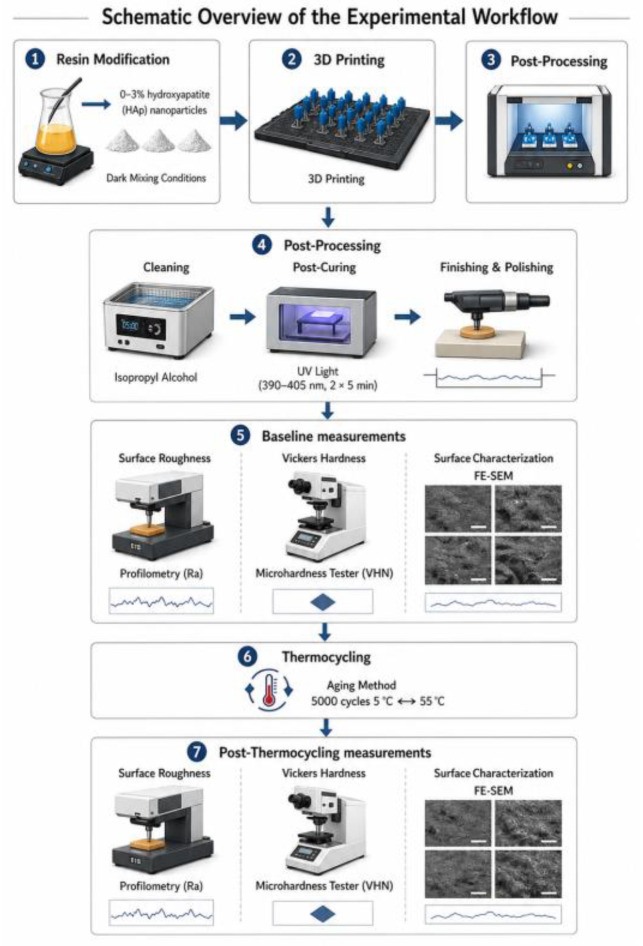
Schematic overview of the experimental workflow, including resin modification, 3D printing, post-processing, thermocycling, and surface and mechanical evaluations.

This in vitro study evaluated the effect of nano-hydroxyapatite (nano-HAp) incorporation on the surface roughness and mechanical properties of a 3D-printed permanent crown resin (Saremco Print CROWNTEC, shade A2; Saremco Dental AG, Rebstein, Switzerland). Nano-HAp (Nanografi Nanotechnology, Ankara, Turkey; purity ≥ 95%, average particle size ≈ 50 nm) was incorporated into the resin at concentrations of 0 wt% (control), 1 wt%, 2 wt%, and 3 wt% (Table 1). These concentrations were selected based on previous studies demonstrating that low-to-moderate nanoparticle loading (1–3 wt%) may enhance mechanical properties without adversely affecting printability or promoting excessive agglomeration [11,12,15]. The overall experimental workflow of the study is illustrated in Figure 1.

A total of 84 disk-shaped specimens were fabricated and allocated into four groups (n = 21 per group) according to nano-HAp concentration. Within each group, 13 specimens were assigned to surface roughness (Ra) measurements, 7 specimens to Vickers hardness (VHN) testing, and 1 specimen to qualitative field-emission scanning electron microscopy (FE-SEM) analysis. The same specimens used for Ra and VHN measurements were evaluated longitudinally before and after thermocycling. Specimens used for FE-SEM analysis were separate from those used for quantitative testing.

The difference in sample size between Ra (n = 13) and VHN (n = 7) measurements was based on the distinct methodological characteristics of each test. Surface roughness measurements are more sensitive to localized surface variability and therefore require a larger sample size to obtain reliable mean values, as reported in previous studies evaluating dental restorative materials [4,25,26]. In contrast, Vickers hardness testing involves multiple indentations per specimen, allowing internal averaging and reducing the number of specimens required without compromising statistical reliability [27]. This approach is consistent with previous investigations on the mechanical and surface properties of dental biomaterials [4,28].

### 2.2. Specimen Fabrication and Nanoparticle Incorporation

Nano-HAp powder was weighed using a precision balance (Precisa XB 220A, Dietikon, Switzerland) and incorporated into the resin at the specified weight percentages. The mixtures were magnetically stirred for 24 h, followed by homogenization for 20 min using a 250 W ultra-homogenizer (Ultra-Turrax, Janke & Kunkel, Staufen, Germany) to promote nanoparticle dispersion. All procedures were performed under controlled lighting conditions using amber containers to minimize premature photopolymerization.

No direct quantitative assessment of nanoparticle dispersion (e.g., transmission electron microscopy) was performed; therefore, conclusions regarding nanoparticle distribution should be interpreted with caution. Nevertheless, similar mixing protocols have been widely used in studies involving nano-hydroxyapatite incorporation into dental materials [13,14,29]. All modified resin formulations remained printable under the selected conditions, with no observable defects such as incomplete curing, delamination, or print failure.

### 2.3. Digital Design and Additive Manufacturing

**Figure 2:**
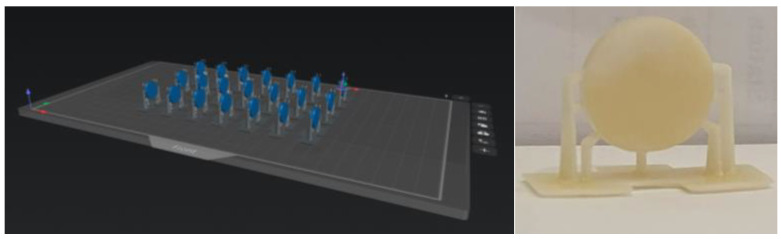
Representative images illustrating the digital design and additive manufacturing of the disc-shaped specimens, including the build platform layout with support structures (**left**) and a printed specimen (control group; 0% nano-HAp) after fabrication (**right**).

Disk-shaped specimens were designed using Blender (Blender Foundation, Amsterdam, The Netherlands; version 4.0), an open-source 3D modeling software, and exported as standard tessellation language (STL) files. The files were processed using slicing software (CHITUBOX, version 1.0.1; Shenzhen CBD Technology Co., Ltd., Shenzhen, China) (Figure 2).

Specimens were fabricated using a masked stereolithography (MSLA) 3D printer (Phrozen Sonic Mighty 12K; Phrozen Technology, Hsinchu, Taiwan). Printing parameters included a layer thickness of 50 µm and a printing orientation of 90°. The bottom layer exposure time was set to 30 s for the initial 6 layers, followed by a normal layer exposure time of 2.5 s. The lifting speed was set to 80 mm/min, and the retract speed to 150 mm/min, with a light-off delay of 1 s. These parameters were selected based on manufacturer-recommended settings for the resin–printer combination and were applied consistently across all specimens.

### 2.4. Post-Processing and Surface Preparation

Following fabrication, specimens were cleaned in an ultrasonic bath containing 96% isopropyl alcohol to remove uncured resin residues and then air-dried. Post-curing was performed using a UV curing unit (Phrozen Cure; Phrozen Technology Co., Ltd., Hsinchu City, Taiwan) for two consecutive cycles of 5 min (total 10 min) within a wavelength range of 390–405 nm, in accordance with the manufacturer’s recommendations. The light intensity was not specified by the manufacturer.

Support structures were removed using a low-speed rotary instrument with a tungsten carbide bur. Surfaces were standardized through sequential polishing using silicon carbide abrasive papers (1000, 1200, and 2000 grit) under continuous water cooling, followed by polishing with a universal polishing paste (Ivoclar Vivadent, Schaan, Liechtenstein), as described in previous studies [25,26]. All specimens were stored in distilled water at 37 °C for 24 h prior to testing.

### 2.5. Surface Roughness (Ra) Measurements

Surface roughness measurements were performed on 13 specimens per group (n = 13) using a contact profilometer (Surtronic 25; Taylor Hobson, Leicester, UK), following established protocols [25]. Three measurements were obtained per specimen at different locations, with a minimum spacing of 2 mm. Measurement parameters included a cutoff length of 0.8 mm, a traverse speed of 0.5 mm/s, and an evaluation length of 7 mm. The mean value was recorded as the Ra for each specimen.

### 2.6. Vickers Hardness (VHN) Measurements

Vickers hardness was measured on 7 specimens per group (n = 7) using a digital microhardness tester (HMV-2 VAS; Shimadzu Corporation, Kyoto, Japan), following standard microhardness testing procedures [27]. Three indentations were made per specimen under a load of 300 g with a dwell time of 10 s. The mean value was recorded as the VHN.

### 2.7. Field-Emission Scanning Electron Microscopy (FE-SEM)

For qualitative surface characterization, one representative specimen per group was analyzed before and after thermocycling. Specimens were selected based on their proximity to group mean Ra values to reflect typical surface characteristics.

Specimens were sputter-coated with a silver–palladium alloy (80% Ag–20% Pd) and examined using a field-emission scanning electron microscope (Gemini 500; Carl Zeiss, Oberkochen, Germany) at an accelerating voltage of 3 kV and a working distance of approximately 4.1 mm. Images were obtained at magnifications of ×1000 and ×2500.

FE-SEM analysis was used exclusively for qualitative surface evaluation. The selected magnifications and surface imaging approach are not sufficient to reliably detect nanoscale nanoparticle dispersion or agglomeration within the resin matrix; therefore, no definitive conclusions regarding nanoparticle distribution were drawn.

### 2.8. Thermocycling Protocol

Artificial aging was simulated using a thermocycling device (SD Mechatronic GmbH, Feldkirchen-Westerham, Germany). Specimens were subjected to 5000 thermal cycles between 5 °C and 55 °C, with a dwell time of 30 s and a transfer time of 10 s. This protocol is widely used to simulate intraoral aging conditions [30,31]. Surface roughness and Vickers hardness were measured before and after thermocycling using the same specimens assigned to each test.

### 2.9. Power Analysis and Statistical Procedures

Sample size was determined a priori using G*Power software (version 3.1.9.7; Heinrich Heine University, Düsseldorf, Germany). The calculation was performed for repeated-measures analysis of variance with a between–within interaction design, using four experimental groups and two repeated measurements. A significance level of 0.05 and statistical power of 80% were applied.

An effect size of f = 0.60 was selected based on the magnitude of differences reported in previous studies investigating nanoparticle-modified dental resin materials and hydroxyapatite-reinforced polymer-based dental materials [11,12,15]. This value was considered appropriate because the present study was expected to detect relatively large changes in surface roughness and hardness after nanoparticle incorporation and thermocycling. The effect size was primarily based on anticipated differences in surface roughness (Ra), which is highly sensitive to changes in filler incorporation, surface composition, and aging procedures.

Because the selected effect size represents a large effect according to Cohen’s criteria, the sample size calculation was interpreted conservatively. It should also be noted that the study was primarily powered to detect main effects of nano-HAp concentration and thermocycling, whereas the detection of interaction effects may require larger sample sizes. Therefore, interaction findings should be interpreted with appropriate caution.

Specimen allocation and measurement sequence were randomized to minimize selection and measurement bias. All measurements were performed by a single calibrated operator. Blinding was not implemented because the experimental groups could be visually distinguished after nano-HAp incorporation.

Statistical analyses were conducted using IBM SPSS Statistics software (version 23.0; IBM Corp., Armonk, NY, USA). Data normality was assessed using the Shapiro–Wilk test. Surface roughness and Vickers hardness were analyzed separately using a two-way mixed-design ANOVA, with nano-HAp concentration as the between-subject factor and thermocycling condition, before versus after thermocycling, as the within-subject factor. Tukey’s HSD test was used for post hoc comparisons. Statistical significance was set at *p* < 0.05.

## 3. Results

### 3.1. Surface Roughness (Ra)

A two-way mixed ANOVA revealed a statistically significant effect of thermocycling on surface roughness (Ra) (F(1,48) = 353.72, *p* < 0.001, η^2^_p_ = 0.88), with all groups demonstrating increased Ra values after thermocycling (Table 2). No statistically significant differences were observed among groups at baseline (F(3,48) = 0.52, *p* = 0.67, η^2^_p_ = 0.03), with mean values ranging from 0.12 ± 0.03 µm to 0.15 ± 0.05 µm.

A significant interaction between nano-HAp concentration and thermocycling was identified (F(3,48) = 24.91, *p* < 0.001, η^2^_p_ = 0.61), indicating that the magnitude of Ra increase differed significantly among groups. Following thermocycling, Ra values increased significantly in all groups (*p* < 0.001 for all within-group comparisons). The control group (0% nano-HAp) exhibited an increase from 0.14 ± 0.03 µm to 0.37 ± 0.06 µm, while the 1%, 2%, and 3% nano-HAp groups increased to 0.56 ± 0.18 µm, 0.81 ± 0.18 µm, and 1.16 ± 0.47 µm, respectively.

**Figure 3:**
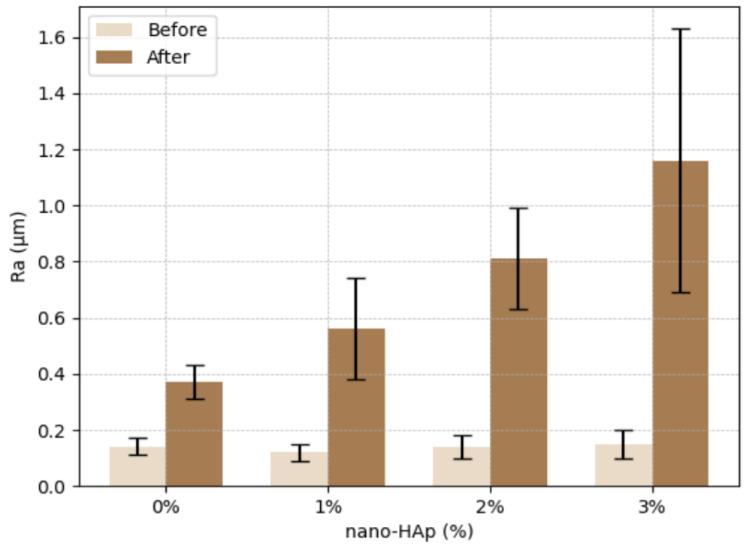
Surface roughness (Ra) values (mean ± standard deviation) of 3D-printed crown resin specimens containing 0%, 1%, 2%, and 3% nano-hydroxyapatite (nano-HAp) before and after thermocycling.

Post hoc Tukey analysis demonstrated statistically significant differences among groups after thermocycling (*p* < 0.001). The 3% nano-HAp group exhibited significantly higher Ra values compared with all other groups, whereas no statistically significant differences were observed between the 0% and 1% groups or between the 1% and 2% groups (Figure 3).

### 3.2. FE-SEM Surface Morphology

**Figure 4:**
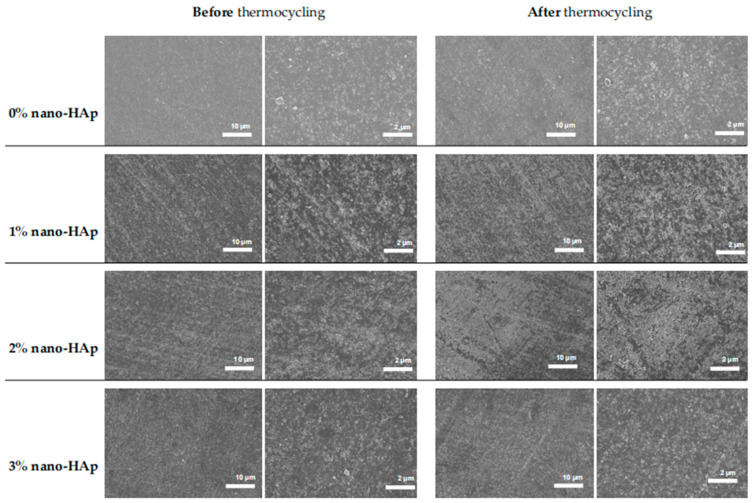
Representative FE-SEM images of the surface morphology of 3D-printed crown resin specimens containing 0% nano-HAp, 1% nano-HAp, 2% nano-HAp, and 3% nano-HAp before and after thermocycling. The first and second column presents the images obtained before thermocycling at ×1000 magnification (scale bar = 10 µm), and at ×2500 magnification (scale bar = 2 µm), respectively. The third and fourth columns presents the images obtained after thermocycling at ×1000 magnification (scale bar = 10 µm), and at ×2500 magnification (scale bar = 2 µm), respectively.

Representative FE-SEM micrographs of the control (0% nano-HAp) and nano-hydroxyapatite (nano-HAp)-modified groups (1–3% nano-HAp) before and after thermocycling are presented in Figure 4. Images were obtained at two magnifications (×1000 and ×2500; scale bars = 10 µm and 2 µm) to assess surface morphology at both micro- and submicrometer levels.

Before thermocycling, all groups exhibited relatively homogeneous surface topographies characterized by polishing-induced linear marks and shallow grooves. At lower magnification (10 µm scale), no discernible differences in surface texture were observed among groups. Higher magnification (2 µm scale) revealed fine micro-irregularities distributed across the surfaces; however, no evidence of nanoparticle agglomeration, microcracking, or structural discontinuities was detected in any group at baseline.

Following thermocycling, surfaces in all groups appeared more heterogeneous. At the 10 µm scale, an increase in surface irregularities and micro-texture was observed, consistent with the quantitative increase in Ra values. These changes were more pronounced in the nano-HAp-modified groups, particularly at higher concentrations. At higher magnification, post-thermocycling images demonstrated an increased density of fine surface features and shallow micro-relief patterns, suggestive of hydrothermal degradation of the resin matrix.

Despite the statistically significant increase in surface roughness measured by profilometry—particularly in the 3% nano-HAp group—FE-SEM analysis did not reveal distinct group-specific defects, extensive nanoparticle agglomeration, or severe microstructural damage. This apparent discrepancy may be attributed to differences in methodological sensitivity, as contact profilometry quantitatively captures micrometer-scale height variations over a broader surface area, whereas FE-SEM provides localized qualitative surface assessment. FE-SEM observations support the profilometric findings by demonstrating thermocycling-induced surface alterations, although these changes remained subtle at the microscale and did not involve gross structural deterioration.

### 3.3. Vickers Hardness (VHN)

A two-way mixed ANOVA demonstrated a significant effect of thermocycling on Vickers hardness (VHN) (F(1,24) = 517.84, *p* < 0.001, η^2^_p_ = 0.96), with all groups showing decreased hardness values after thermocycling (Table 3). A statistically significant effect of nano-HAp concentration was also observed (F(3,24) = 4.92, *p* = 0.008, η^2^_p_ = 0.38).

In addition, a significant interaction between nano-HAp concentration and thermocycling was identified (F(3,24) = 3.27, *p* = 0.039, η^2^_p_ = 0.29), indicating that the magnitude of hardness reduction differed among groups. At baseline, VHN values differed significantly among groups (*p* = 0.041), with mean values ranging from 39.73 ± 8.35 in the control group (0% nano-HAp) to 49.26 ± 4.66 in the 3% nano-HAp group.

Following thermocycling, VHN values decreased significantly in all groups (*p* < 0.001 for all within-group comparisons). The control group showed a reduction from 39.73 ± 8.35 to 20.69 ± 2.27, while the 1% and 2% nano-HAp groups decreased to 15.50 ± 2.01 and 17.33 ± 1.28, respectively. The 3% nano-HAp group exhibited the highest post-thermocycling VHN values (24.66 ± 1.51).

**Figure 5:**
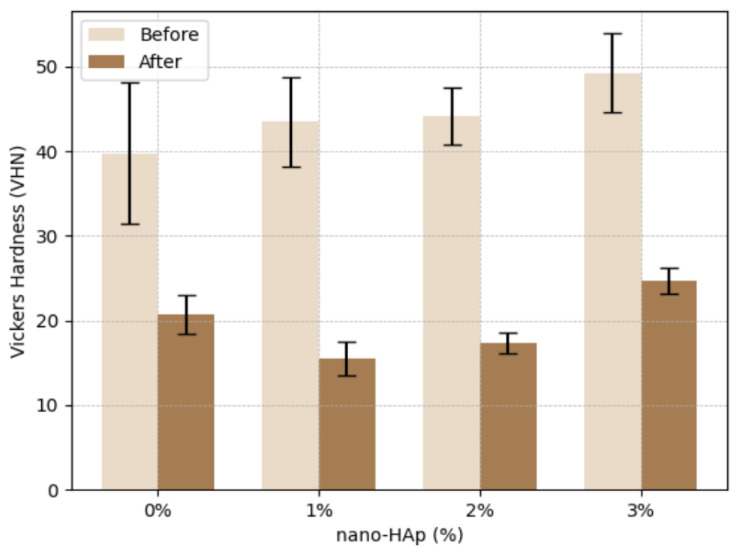
Vickers hardness (VHN) values (mean ± standard deviation) of 3D-printed crown resin specimens containing 0%, 1%, 2%, and 3% nano-hydroxyapatite (nano-HAp) before and after thermocycling.

Post hoc Tukey analysis revealed that the 3% nano-HAp group exhibited significantly higher VHN values compared with the 0%, 1%, and 2% groups, whereas no statistically significant difference was observed between the 1% and 2% nano-HAp groups (*p* < 0.001) (Figure 5).

## 4. Discussion

The present study investigated the influence of hydroxyapatite nanoparticle (nano-HAp) incorporation at concentrations of 1–3 wt% on the surface roughness (Ra) and Vickers hardness (VHN) of a 3D-printed permanent crown resin before and after thermocycling. This assessment is clinically relevant, as additive manufacturing technologies are increasingly integrated into restorative dentistry for definitive prosthetic applications, where long-term mechanical stability and surface integrity are critical for functional performance, maintenance, and biological compatibility. Within the limitations of this in vitro study, thermocycling resulted in a statistically significant increase in Ra across all groups and a significant reduction in VHN. Although the 3 wt% nano-HAp group exhibited the highest post-aging hardness values, this finding should be interpreted cautiously due to baseline differences among groups. Overall, nano-HAp incorporation influenced the aging response of 3D-printed crown resins, with surface roughness and hardness demonstrating competing effects.

Additive manufacturing has become a central component of modern digital dentistry due to its efficiency, reduced material waste, and ability to fabricate complex geometries with high precision [2]. These advantages have enabled its application in the fabrication of dental models, surgical guides, provisional and definitive restorations, and implant-supported prostheses [2,10]. However, despite its growing clinical adoption, concerns remain regarding the long-term performance of 3D-printed materials, particularly in terms of their mechanical properties and resistance to aging [3,10]. Studies comparing printed and conventionally manufactured restorations emphasize that performance is strongly dependent on material composition, printing parameters, and post-processing protocols [3,5,32]. Compared with conventionally milled CAD/CAM materials, which typically exhibit higher initial hardness and superior wear resistance, the nano-modified 3D-printed resins evaluated in this study still demonstrate lower absolute mechanical performance, highlighting the need for further optimization of additive materials for long-term clinical use.

Surface roughness is a critical parameter influencing the clinical success of restorative materials, as it directly affects plaque accumulation, bacterial adhesion, wear behavior, and esthetic outcomes [6,7,8,9,26,28,30]. In the present study, baseline Ra values did not differ significantly among groups, indicating that the standardized finishing and polishing procedures were effective in producing comparable initial surface topographies. This finding is consistent with previous studies demonstrating that finishing protocols can significantly influence surface characteristics and may mask intrinsic material differences at baseline [26,28]. The absence of baseline differences also suggests that nano-HAp incorporation, under the present preparation conditions, did not introduce gross surface irregularities or defects prior to aging.

Following thermocycling, Ra values increased significantly in all groups. Notably, Ra values increased from approximately 0.12–0.15 µm at baseline to 0.37 µm in the control group and up to 1.16 µm in the 3 wt% group after thermocycling, representing a substantial and clinically relevant increase in surface roughness. Importantly, all post-thermocycling Ra values exceeded the clinically accepted threshold of approximately 0.2 µm associated with increased bacterial adhesion, suggesting that additional surface treatments may be required for clinical application. Although a general increasing trend with nano-HAp concentration was observed, this pattern was not strictly stepwise, as lower concentrations (1 wt% and 2 wt%) did not differ significantly from adjacent groups. These findings led to the rejection of the second null hypothesis, as thermocycling significantly increased surface roughness across all groups. Additionally, the first null hypothesis was rejected, as nano-HAp concentration influenced Ra values after thermocycling. Thermocycling is widely used to simulate intraoral thermal fluctuations and has been shown to induce hydrothermal degradation in polymer-based restorative materials [30,31]. The observed increase in Ra is consistent with previous reports indicating that thermal stress and water sorption can alter the polymer matrix, leading to surface degradation and increased roughness [30]. In 3D-printed resins, these effects may be further influenced by factors such as layer-by-layer fabrication, degree of conversion, and post-curing efficiency [3,31].

The increase in Ra following aging suggests that nano-HAp incorporation influences the material’s response to thermal stress rather than its baseline surface characteristics. This phenomenon may be explained by the increased number of filler–matrix interfaces associated with higher nanoparticle concentrations, which can facilitate water diffusion and promote localized microstructural degradation during thermocycling [11,12,29]. The high surface area of nano-HAp particles further enhances interfacial interactions, potentially affecting stress distribution and hydrolytic stability within the resin matrix [17,29]. Additionally, even minor variations in nanoparticle dispersion may become more pronounced after aging, contributing to the observed increase in surface irregularities [11,12,29].

From a clinical perspective, increased surface roughness is undesirable, as it has been associated with enhanced bacterial adhesion, plaque accumulation, and periodontal inflammation [6,7]. Moreover, rougher surfaces may compromise esthetics and accelerate material wear [8,9]. Therefore, while nano-HAp incorporation may offer potential mechanical benefits, its impact on surface properties must be carefully considered. In this context, additional finishing procedures, surface coatings, or glazing techniques may be necessary to reduce surface roughness below clinically acceptable thresholds following aging.

Vickers hardness is a key mechanical property reflecting the resistance of materials to indentation, abrasion, and wear [4,27,30]. In the present study, thermocycling resulted in a significant reduction in VHN across all groups, indicating a softening effect associated with hydrothermal aging. This finding supports the rejection of the second null hypothesis, as thermocycling significantly reduced Vickers hardness across all groups. This observation is consistent with previous studies demonstrating that exposure to temperature fluctuations and moisture can reduce the mechanical performance of polymer-based dental materials [27,30]. The reduction in hardness may be attributed to moisture-induced plasticization and relaxation of the polymer network, which increases chain mobility and decreases resistance to deformation [30,31].

Although the 3 wt% nano-HAp group exhibited the highest post-thermocycling VHN values, it is important to note that this group also showed the highest baseline hardness. Therefore, these findings should be interpreted as differences in absolute hardness values rather than true retention, indicating that the 3 wt% formulation maintained the highest hardness after aging. In contrast, the 1 wt% and 2 wt% nano-HAp groups demonstrated lower post-aging hardness values compared with the control group, suggesting that lower filler concentrations may not provide sufficient reinforcement and may even negatively influence mechanical performance under aging conditions. These findings support the rejection of the first null hypothesis, as nano-HAp concentration significantly influenced Vickers hardness, particularly in terms of absolute post-thermocycling values. The significant interaction effect indicates that the influence of nano-HAp concentration on hardness is dependent on thermocycling conditions, with higher concentrations resulting in higher post-aging hardness values compared with lower concentrations. However, due to the presence of baseline differences, this interaction should be interpreted as a differential response pattern rather than definitive evidence of enhanced aging resistance. These findings led to the rejection of the third null hypothesis, indicating that the effect of nano-HAp concentration on Vickers hardness was dependent on thermocycling conditions. Future studies incorporating extended aging cycles and combined mechanical wear simulation are required to better evaluate long-term performance under clinically relevant conditions.

The relatively higher hardness values observed in the 3 wt% group may be attributed to the increased contribution of the inorganic filler phase during indentation, particularly as the polymer matrix becomes more susceptible to degradation after aging [12,29,31]. However, this mechanical advantage was accompanied by a greater increase in surface roughness, highlighting the presence of competing effects between mechanical reinforcement and surface integrity [12,26,28]. Previous studies have also demonstrated that improvements in one material property do not necessarily correspond to improvements in others, emphasizing the need for a balanced approach in material design [4,28].

FE-SEM analysis revealed similar surface morphologies across all groups before and after thermocycling, with surfaces characterized by polishing-induced grooves and linear features. No obvious evidence of nanoparticle agglomeration, microcracking, or structural discontinuities was observed at the examined magnifications. However, this observation should be interpreted cautiously, as FE-SEM imaging of limited surface areas cannot exclude the presence of nanoscale clustering, subsurface heterogeneity, or localized dispersion defects.

After thermocycling, FE-SEM images demonstrated a modest increase in surface heterogeneity and micro-texture, particularly in nano-HAp-modified groups. However, these changes remained subtle and did not manifest as large-scale surface damage. The discrepancy between the significant increase in Ra values and the relatively minor changes observed in SEM images can be explained by methodological differences. Contact profilometry captures cumulative surface height variations over a larger area and is highly sensitive to micrometer-scale changes, whereas FE-SEM provides localized qualitative imaging that may not detect subtle but widespread surface alterations.

In addition, the presence of polishing-induced grooves observed in SEM images may have influenced profilometric measurements after thermocycling, as these pre-existing features could act as sites for localized degradation or amplify roughness readings. Furthermore, nanoscale clustering or interfacial heterogeneities cannot be excluded and may contribute to the observed changes in surface roughness and hardness, despite not being clearly visible in SEM images. Despite these observations, it should be emphasized that, given the limited sampling (one specimen per group) and the inherent constraints of SEM imaging, these findings should be considered qualitative and illustrative rather than definitive, and do not allow conclusions regarding nanoparticle dispersion or interfacial mechanisms.

The use of non-silanized nano-HAp in this study allowed evaluation of the material behavior without chemical coupling enhancement; however, this approach also alters filler–matrix interactions and may contribute to increased surface roughness and suboptimal mechanical performance compared with silanized or surface-modified nanoparticles. Therefore, the present findings should be interpreted as reflecting a non-optimized system rather than the full potential of nano-HAp-reinforced materials. Future investigations should evaluate surface-modified nanoparticles and assess ion release behavior, as calcium and phosphate leaching may influence both structural stability and long-term biocompatibility.

This study has several limitations. Thermocycling alone does not fully replicate the complexity of the oral environment, where mechanical loading, chemical exposure, and biofilm activity may influence material behavior. Future studies should incorporate extended aging protocols (e.g., higher thermocycling cycle numbers) as well as combined mechanical wear simulations to better approximate clinical conditions and evaluate long-term material performance. Additionally, only one resin system was evaluated, limiting the generalizability of the findings. The use of non-silanized nano-HAp may have influenced interfacial behavior, and only a limited concentration range was investigated.

## 5. Conclusions

Within the limitations of this in vitro study, the following conclusions can be drawn:

1. Nano-HAp incorporation did not influence baseline surface roughness, as no statistically significant differences in Ra were observed among groups prior to thermocycling, indicating comparable initial surface conditions following standardized finishing and polishing.
2. Thermocycling resulted in a significant increase in surface roughness in all groups, with post-aging Ra values (0.37–1.16 µm) exceeding the clinically accepted threshold of approximately 0.2 µm associated with increased bacterial adhesion, indicating that additional surface finishing, polishing, or coating procedures are required to achieve clinically acceptable surface characteristics after aging.
3. Vickers hardness decreased significantly after thermocycling in all groups, confirming the susceptibility of the tested material to hydrothermal degradation. The 3 wt% nano-HAp group exhibited the highest post-aging hardness values; however, this reflects higher absolute values rather than improved retention, as baseline hardness also differed among groups.
4. Lower nano-HAp concentrations (1 wt% and 2 wt%) did not improve mechanical performance and showed reduced post-aging hardness compared with the control, indicating that limited filler addition may not provide effective reinforcement under aging conditions.
5. FE-SEM observations revealed generally similar surface morphologies across groups, with modest increases in surface heterogeneity after thermocycling. However, these qualitative observations should be interpreted cautiously, as the imaging approach was limited in sampling and may not capture subtle or localized surface changes.
6. Overall, nano-HAp incorporation produced a trade-off between mechanical and surface properties, with higher concentrations associated with increased post-aging hardness but also greater surface roughness. As a result, none of the tested formulations simultaneously satisfied both mechanical and surface requirements under the present aging conditions.
7. The findings are limited to the investigated concentration range (1–3 wt%) and the use of non-silanized nanoparticles. Future work should explore a broader formulation window, including surface-modified fillers and optimized post-processing strategies, to achieve a better balance between mechanical performance and surface stability.

## Figures and Tables

**Table 1:** A summary of the materials used in this study.

Material Group	Commercial Name	Manufacturer	Composition/Ingredients
Permanent Crown Resin	Saremco Print CROWNTEC	Saremco Dental AG, Rebstein, Switzerland	Esterification products of 4.4′-isopropylidiphenol, ethoxylated and 2-methylprop-2enoic acid, silanized dental glass, pyrogenic silica, initiators, inorganic fillers (particle size ≈ 0.7 μm) accounts for 30–50 wt%
Nanoparticles	Hydroxyapatite Nanoparticles	Nanografi Nanotechnology, Ankara, Turkey	Hydroxyapatite nanoparticles purity 95% min. Average particle size ≈ 50 nm; Ca ≤ 27.4%, P ≤ 12.5%; pH (1.0% aqueous solution) = 6.95; 0.3% Na, 0.035% Al, 0.05% Fe, 0.048% SO_4_

wt% = Weight percentage; μm = Micrometer; Ca = Calcium; P = Phosphorus; Na = Sodium; Al = Aluminum; Fe = Iron; SO_4_ = Sulfate.

**Table 2:** Surface roughness (Ra) values (mean ± SD) before and after thermocycling according to nano-HAp concentration.

Nano-HAp (%)	Before (Ra, µm)	After (Ra, µm)
0	0.14 ± 0.03 ^a^	0.37 ± 0.06 ^a^
1	0.12 ± 0.03 ^a^	0.56 ± 0.18 ^a^^b^
2	0.14 ± 0.04 ^a^	0.81 ± 0.18 ^b^
3	0.15 ± 0.05 ^a^	1.16 ± 0.47 ^c^

Values are presented as mean ± standard deviation. Different superscript letters within each column indicate statistically significant differences between groups (Tukey HSD, *p* < 0.05).

**Table 3:** Vickers Hardness (VHN) Values (Mean ± SD) before and after thermocycling according to nano-HAp concentration.

Nano-HAp (%)	Before (VHN)	After (VHN)
0%	39.73 ± 8.35 ^a^^b^	20.69 ± 2.27 ^b^
1%	43.46 ± 5.35 ^b^	15.50 ± 2.01 ^a^
2%	44.19 ± 3.37 ^b^	17.33 ± 1.28 ^a^^b^
3%	49.26 ± 4.66 ^c^	24.66 ± 1.51 ^c^

Values are presented as mean ± standard deviation. Different superscript letters within each column indicate statistically significant differences between groups (Tukey HSD, *p* < 0.05).

## Data Availability

The original contributions presented in the study are included in the article; further inquiries can be directed to the corresponding author.

## Abbreviations

The following abbreviations are used in this manuscript:

CAD/CAM	Computer-Aided Design/Computer-Aided Manufacturing
3D	Three-Dimensional
MSLA	Masked Stereolithography
nano-HAp	Nano-Hydroxyapatite
HAp	Hydroxyapatite
Ra	Surface Roughness
VHN	Vickers Hardness Number
FE-SEM	Field-Emission Scanning Electron Microscopy
UV	Ultraviolet
STL	Standard Tessellation Language
